# Radiological Explorations of Patients with Upper or Febrile Urinary Tract Infection

**DOI:** 10.3390/idr16020015

**Published:** 2024-02-23

**Authors:** Katia Vanolli, Mike Libasse Jost, Olivier Clerc, Daniel Genné, Gregor John

**Affiliations:** 1 Department of Internal Medicine, Neuchâtel Hospital Network, Rue de la Maladière 45, CH-2000 Neuchâtel, Switzerland; katia.vanolli@rhne.ch (K.V.);; 2 Department of Internal Medicine, Bienne Hospital Center, Chante-Merle 84, CH-2501 Bienne, Switzerlanddaniel.genne@szb-chb.ch (D.G.); 3 Department of Medicine, Geneva University, Michel-Servet 1, CH-1206 Geneva, Switzerland; 4 Department of Internal Medicine, Geneva University Hospitals (HUG), Gabrielle-Perret-Gentil 4, CH-1205 Geneva, Switzerland

**Keywords:** acute pyelonephritis, urinary tract infection, radiological imaging, guideline adherence, predictive factors

## Abstract

Recent European Association of Urology (EAU) guidelines and a clinical prediction rule developed by Van Nieuwkoop et al. suggest simple criteria for performing radiological imaging for patients with a febrile urinary tract infection (UTI). We analysed the records of patients with a UTI from four hospitals in Switzerland. Of 107 UTI patients, 58% underwent imaging and 69% (95%CI: 59–77%) and 64% (95%CI: 54–73%) of them were adequately managed according to Van Nieuwkoop’s clinical rule and EAU guidelines, respectively. However, only 47% (95%CI: 33–61%) and 57% (95%CI: 44–69%) of the imaging performed would have been recommended according to their respective rules. Clinically significant imaging findings were associated with a history of urolithiasis (OR = 11.8; 95%CI: 3.0–46.5), gross haematuria (OR = 5.9; 95%CI: 1.6–22.1) and known urogenital anomalies (OR = 5.7; 95%CI: 1.8–18.2). Moreover, six of 16 (38%) patients with a clinically relevant abnormality displayed none of the criteria requiring imaging according to Van Nieuwkoop’s rule or EAU guidelines. Thus, adherence to imaging guidelines was suboptimal, especially when imaging was not recommended. However, additional factors associated with clinically significant findings suggest the need for a new, efficient clinical prediction rule.

## 1. Introduction

Acute pyelonephritis (APN) is a common infection, with an estimated yearly incidence in the United States of 1.2–1.3‰ in women outpatients, 0.2–0.3‰ in men outpatients, 0.3–0.4‰ in women inpatients and 0.1–0.2‰ in men inpatients [1,2]. Urinary tract infections (UTIs) rank as the ninth most common reason for an emergency department (ED) visit in the United States [1,2]. The overall annual financial burden of APN is estimated at USD 2.1 billion [3]. 

To exclude APN complications such as urinary stones and other causes of obstruction, clinicians must decide whether to perform a computerised tomography (CT) scan or an ultrasound (US) scan. Although there is a consensus that not all APN patients will benefit from radiological imaging [4], identifying those who will remains challenging. A total of 52–87% of APN or febrile UTI patients undergo imaging depending on the setting [5,6,7,8,9,10]. Although the estimated global incidence of urolithiasis was 4.4 million in 2019, the proportion of urolithiasis-associated APN or other complications that may require urgent intervention remains largely unknown [11]. CT is the most sensitive scanning method for most complications, whereas US should be considered as an initial imaging option given its wider availability, safety and lower cost [12,13].

Van Nieuwkoop et al. proposed simple criteria for deciding which patients should undergo upper urinary tract imaging [10]. According to their study, the absence of three criteria (previous renal stones, high urine pH and renal insufficiency) should exclude a clinically significant urinary tract abnormality with a negative predictive value of 93%, which could reduce the need for imaging in cases of febrile UTIs by up to 40%. However, this study lacked external validity [14] and did not consider clinical instability. Overlooking urinary obstruction in severely ill patients (with sepsis or in shock) has been shown to increase the risk of unfavourable outcomes [15]. The European Association of Urology (EAU) 2023 guidelines listed recommendations for imaging in cases of uncomplicated APN and urosepsis [16]. They added two criteria to Van Nieuwkoop’s clinical rules for when imaging should be considered: persistent fever after treatment initiation (72 h) and sepsis. However, the impact of these recommendations on daily practice, especially in EDs, has been little explored.

We aimed to calculate the proportion of urinary tract imaging performed on APN patients admitted through EDs and compliance with Van Nieuwkoop’s clinical rule or EAU guideline recommendations. We also aimed to reveal factors associated with clinically significant abnormal imaging findings that require active intervention.

## 2. Materials and Methods

We performed a retrospective analysis on a cohort of patients with APN who had previously been enrolled prospectively [17]. Data collection had already been approved by all four participating hospitals’ ethics committees. The present article follows the STROBE checklist for reporting on observational studies [18].

### 2.1. Design, Setting and Participants 

All consecutive patients with an upper UTI diagnosed in the EDs of four mid-sized secondary hospitals in western Switzerland between February 2019 and June 2021 were eligible. Inclusion criteria were being ≥18 years old, having a urinary complaint, clinical indications of an upper UTI (febrile condition, chills, flank pain or nausea/vomiting) and leukocyturia. 

Exclusion criteria were the presence of an indwelling urinary catheter inserted for more than 24 h; antibiotic therapy in the week preceding inclusion, anuria, urological or gynaecological surgery in the last six weeks; pregnancy; a severe allergic reaction to penicillin or cephalosporin; and an inability to give informed consent. We also excluded patients without a final diagnosis of upper UTI.

All physicians used a standardised questionnaire to avoid measurement bias. 

### 2.2. Outcomes and Measurements

Our primary outcome was the proportion of patients correctly managed (relative to imaging) according to recommendations. Secondary outcomes were the proportion of clinically significant urological disorders found on imaging, plus any associated factors and their predictive performance. 

Imaging performed during management (US, CT and other methods) was dichotomised as ‘initial’ (performed within 24 h of admission) or ‘delayed’ (performed more than 24 h after admission). Only abdominal/urinary imaging ordered to exclude UTI complications, obstructions or other infectious sources were considered. Elective imaging for other reasons (e.g., oncological extension or follow-up images) was not considered. 

Urinary imaging results were dichotomised into ‘clinically relevant’ and ‘clinically irrelevant’ categories, as per Van Nieuwkoop’s study (Table S1) [6]. Clinically irrelevant findings were further categorised into ‘normal’, ‘minimal urological findings’ and ‘incidental non-urological findings’. Clinically relevant findings were dichotomised into ‘urgent urological disorders’ (e.g., renal abscess, pyonephrosis, obstructive urinary tract stones or any other urinary obstructions accompanying a UTI) and ‘non-urgent urological disorders’ when patients required a non-urgent intervention in the ED or as part of their current hospitalisation. 

We used two clinical rules to classify patient management as appropriate. Firstly, we used the original three-point rule for performing initial imaging (within 24 h) based on Van Nieuwkoop’s study: a history of urolithiasis, urine pH ≥ 7.0 and renal insufficiency (estimated glomerular filtration rate ≤40 mL/min/1.73 m^3^). Secondly, we referred to EAU guidelines for initial and delayed urinary imaging that add two criteria to the initial rules: sepsis/haemodynamic instability and delayed clinical resolution (fever > 72 h). Estimated glomerular filtration was based on the modification of diet in renal disease (MDRD) formula [19]. Haemodynamic instability was defined as any low blood pressure (mean pressure < 65 mmHg, systolic blood pressure < 90 mmHg, or a shock index < 1 and systolic blood pressure < 100 mmHg) and/or skin mottling. Congenital urological malformation, a neurogenic bladder, benign prostatic hyperplasia, urinary cancer and urogenital prolapse were classified as structural urogenital anomalies.

Data on population characteristics, laboratory values, microbiological results and imaging reports were collected during the initial study or, when missing, were retrospectively extracted from patients’ medical charts and hospital databases. Clinical resolution was the first day without fever. There was no follow-up of participants beyond their ED or hospital discharge. 

### 2.3. Statistical Analysis 

The sample size calculation was determined and described in a previous study. It aimed to detect a 10% difference in matched urinary culture growth before and after a first dose of antibiotics, with a study power of 80% and a 0.05 significance level [17]. A safety margin of 100 patients was set to account for potential information loss. 

For the primary analysis, we calculated the proportion of patients managed according to recommendations (with imaging when recommended and without imaging when not recommended) and the 95% confidence interval (95%CI). Patients with missing data on urine pH or renal function were not included in the analysis, but two sensitivity analyses were performed. The first analysis put all patients with missing data into the ‘imaging not recommended’ group, and the second put them into the ‘imaging recommended’ group. 

Associations between clinically relevant abnormal findings found on imaging and clinical factors were explored using logistic regressions. Men requiring a urinary catheter in the ED for acute urinary retention were categorised in the ‘clinically significant imaging’ group, regardless of whether imaging was performed or not. Patients who did not undergo imaging and who had no adverse event during clinical management (requiring an antibiotic change or urinary catheterisation, or leading to delayed fever resolution, ICU admission or hospital death) were considered to have normal/minimal radiological findings (if imaging had been performed). Patients who did not undergo imaging and who had any adverse event during hospitalisation were not included in the analysis. We performed a sensitivity analysis restricted to patients who underwent imaging. We computed sensitivity (Se), specificity (Sp), positive and negative predictive value (PPV and NPV) and the area under the receiver operating characteristic curve (AUC) for each factor. Clinical factors were chosen based on previous studies [5,6,10,20,21]. 

For descriptive statistics, we presented continuous data as medians and interquartile ranges (IQRs) and categorical data as numbers and percentages. We used the chi-squared test, Fisher’s exact test and Kruskal–Wallis test to appropriately compare different groups. Significance levels were set at 5%, and all analyses were performed using STATA software, version 17.0 (StataCorp LP, College Station, TX, USA).

## 3. Results

**Figure 1:**
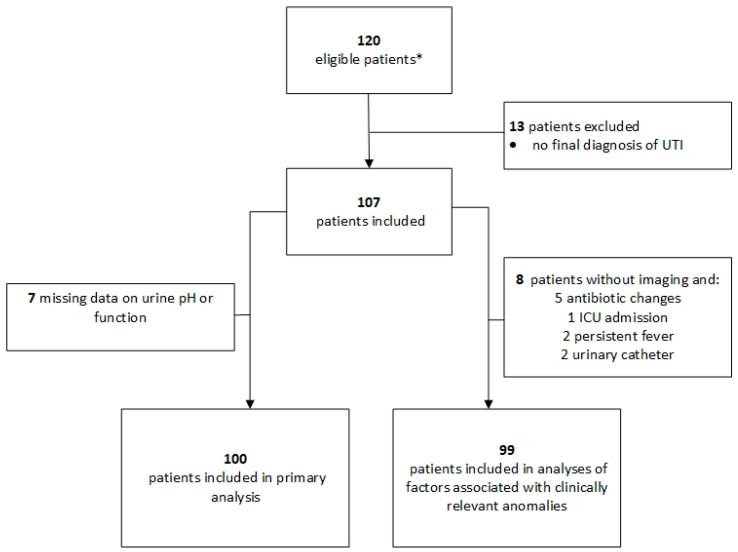
Patient inclusion flowchart. * A total of 17 patients ineligible for the initial study (9 under antibiotics at admission and 8 without pre-antibiotic culture) were added to the 103 patients included in the initial study [17].

The analyses included 107 patients with upper or febrile UTI (Figure 1), with 62 (58%) undergoing upper urinary tract imaging—51 (48%) within the initial management period (24 h) and 11 (10%) later in their hospital stay. The 62 patients underwent 66 upper urinary tract imaging investigations, most frequently a US examination (41/66; 62%), sometimes a CT scan (23/66; 35%) and rarely other methods (2/66; 3%). Patients who underwent upper urinary tract imaging more frequently had a history of kidney stones and reduced kidney function but less frequently a history of neurological disease (Table 1).

### 3.1. Compliance with Clinical Rules and Guidelines (Primary Outcome)

Seven patients had missing data on urinary pH or creatininemia (Figure 1). Overall, 69/100 patients (69%; 95%CI: 59–77%) were managed adequately according to Van Nieuwkoop’s rules, as were 64 (64%; 95%CI: 54–73%) according to EAU recommendations. Compliance was better when clinical rules recommended performing imaging and worse when imaging was not recommended (Table 2). Only 47% (95%CI: 33–61%) of initial imaging and 57% (95%CI: 44–69%) of all the imaging performed (initial and delayed) would have been recommended based on Van Nieuwkoop’s rules and EAU guidelines taken together. 

The proportion of adequately managed patients did not change significantly in the sensitivity analyses, treating all patients with missing variables as either ‘guideline recommended’ or ‘guideline not recommended’. The proportions were 66% (95%CI: 57–75%) and 69% (95%CI: 60–77%), respectively, with Van Nieuwkoop’s rules and 62% (95%CI: 52–70%) and 64% (95%CI: 55–73%), respectively, with EAU recommendations.

### 3.2. Abnormal Findings on Imaging and Associated Factors 

In 15 patients (24%), upper urinary tract imaging found clinically significant anomalies, with 8 patients (13%) requiring urgent urological treatment and 7 patients (11%) requiring non-urgent treatment (Table S1). Nine patients (15%) had urinary stones, which were obstructive in four (6%). One patient who required urgent urinary catheterisation in the ED due to acute urinary retention underwent no imaging and was added to the ‘clinically relevant’ category.

Potential associated factors were tested among the 99 patients who underwent imaging or had an uneventful clinical pathway (Figure 1). Clinically relevant anomalies were associated with Van Nieuwkoop’s rules, a history of gross haematuria, a history of kidney stones and the presence of structural urogenital anomalies (Table 3, Figure S1). Except for a history of kidney stones, the other individual criteria from Van Nieuwkoop’s rules and EAU recommendations were not statistically associated with clinically relevant anomalies. Furthermore, 6 of 16 (38%) patients with clinically relevant findings on imaging presented with none of the criteria for undergoing imaging according to Van Nieuwkoop’s rules or EAU criteria (Table S1).

In the sensitivity analysis restricted to the 62 patients who underwent imaging (Table S2), the association persisted for a history of gross haematuria (OR = 5.9; 95%CI: 1.6–22.1), a history of kidney stones (OR = 11.8; 95%CI: 3.0–46.5) and the presence of structural urogenital anomalies (OR = 5.7; 95%CI: 1.8–18.2). However, the association was not statistically significant for Van Nieuwkoop’s rules (OR = 2.7; 95%CI: 0.8–8.7). 

## 4. Discussion

More than half of febrile UTI patients underwent radiological imaging, although few met the criteria in existing clinical recommendations for performing imaging. However, although Van Nieuwkoop’s rules and EAU guidelines propose straightforward items to aid decisions on imaging, 38% of the patients with clinically significant imaging in our study would have been overlooked if their criteria had been adhered to strictly. Furthermore, we identified other factors that were associated with positive results on imaging and that could add information for a new predictive rule.

The proportion of clinically significant imaging found in our study remained low (14%) considering the total population, but it was similar to the proportion found by Van Nieuwkopp et al. in their derivation cohort. Clinically significant imaging ranges from 7 to 46% across studies but with considerable disparities in populations, diagnosis definitions and the proportions and types of imaging performed [5,6,7,8,9,10,14,20,21,22]. The varying prevalence of urolithiasis across the world (e.g., 5–9% in Europe, 7–13% in North America and 10% in Japan) could influence local practices and explain some of the different proportions of pathological imaging [23].

To the best of our knowledge, only one previous study has assessed the external validity of Van Nieuwkoop’s clinical prediction rule. It showed low NPVs for imaging with clinically relevant anomalies (71%) and for urgent urological anomalies (80%) [14], raising concerns about the reliability of Van Nieuwkoop’s criteria. That study also had several flaws, notably a highly selective patient group (all underwent CT scanning), a setting with a high prevalence of renal stones and a small sample size [14]. Despite the lack of external validation for Van Nieuwkoop’s rule, there is a rationale for developing a better understanding and targeting of patients needing imaging. That rationale aims to mitigate the consequences of any delayed management of complications [15], reduce costs, diminish the risks of nephrotoxic and allergic reactions to iodinated contrast agents, better allocate resources and reduce the energy and environmental costs associated with CT [24,25]. It is also estimated that abdomen–pelvis CT scans are responsible for 1 to 4 radiation-induced cancers per 1000 20-year-old women who undergo them, depending on the multi-phase acquisition and contrast used [26]. 

A history of urolithiasis, gross haematuria and the presence of structural urogenital anomalies were associated with clinically significant anomalies on imaging. Except for a history of urolithiasis, there were no statistically significant associations with other individual Van Nieuwkoop or EAU criteria. Several factors have been associated with the imaging of urological abnormalities in other studies, including impaired renal function (elevated creatinine/blood urea nitrogen or decreased glomerular filtration rate), diabetes mellitus, inflammatory biomarkers and age [5,6,9,20,21,27]. Those studies also identified pain requiring opioid administration, hypotension, a history of uropathy, urinary leucocytosis, a known urinary anomaly, known kidney disease, flank pain and a Pitt score > 1. This range of factors associated with significant urological abnormalities highlights the challenges in identifying clear criteria for an indication for imaging among patients with febrile UTI/APN. 

The present study had some limitations. Firstly, the retrospective analysis meant there were some missing data, even after examination of the hospital charts. We were also unable to calculate sequential organ failure assessment scores to define sepsis. We thus considered signs of haemodynamic instability as indicative of sepsis. Because this definition is more restrictive, a greater number of our patients may have met EAU guideline criteria for imaging. Secondly, our small sample size created a lack of statistical power. Thirdly, the lack of post-discharge follow-up curtailed our ability to assess patients’ short- and medium-term outcomes (notably for those patients who did not undergo imaging). Finally, although this was a multi-centre study, its generalisability may be limited. The prevalence of lithiasis varies worldwide, being notably higher in North America and Japan, which may lead to an increased need for imaging procedures in those regions. 

## 5. Conclusions

In conclusion, our study revealed poor adherence to the appropriate clinical rules on the need to perform imaging for patients with febrile UTI/APN, especially when imaging would not have been recommended otherwise. Apart from a history of urolithiasis, gross haematuria and structural urological anomalies, we found no statistically significant associations between other clinical predictors, the criteria for performing imaging and the significant urinary abnormalities found in that imaging. Overall, these results highlight the need for a new, more efficient clinical prediction rule in cases involving a febrile UTI. 

## Figures and Tables

**Table 1:** Population characteristics.

Characteristics	Total Population *n* = 107	Patients with Radiological Imaging *n* = 62	Patients without Radiological Imaging *n* = 45	*p*-Value
Women, *n* (%)	81 (76%)	45 (73%)	36 (80%)	0.49
Age, y, median (IQR, 25–75%)	54 (34–79)	54 (35–78)	54 (34–83)	0.98
Body mass index, median (IQR, 25–75%)	26 (22–30)	26 (24–31)	23 (21–29)	0.24
Living in a nursing home, *n* (%)	4 (4%)	2 (3%)	2 (5%)	0.99 *
Comorbid conditions, general, *n* (%):				
Cardiovascular	33 (33%)	22 (38%)	11 (26%)	0.19
Neurological	12 (12%)	3 (5%)	9 (21%)	0.03 *
Pulmonary	12 (12%)	7 (12%)	5 (12%)	0.99 *
Digestive	15 (15%)	7 (12%)	8 (19%)	0.40 *
Diabetes	12 (12%)	8 (14%)	4 (9%)	0.55 *
Rheumatological	5 (5%)	3 (5%)	2 (5%)	0.99 *
Oncological disease	9 (9%)	5 (9%)	4 (9%)	0.99 *
Charlson comorbidity index, median (IQR, 25–75%)	0 (0–2)	0 (0–2)	0 (0–2)	0.94
Urogynaecological history				
Any comorbid urological or gynaecological conditions, *n* (%)	30 (30%)	20 (34%)	10 (23%)	0.22
Malformation	4 (4%)	4 (7%)	0 (0%)	0.13 *
Urinary incontinence	7 (7%)	4 (7%)	3 (7%)	0.99 *
Neurogenic bladder	3 (3%)	1 (2%)	2 (5%)	0.57 *
Overactive bladder syndrome	5 (5%)	3 (5%)	2 (5%)	0.69 *
Benign prostatic hyperplasia	5 (5%)	3 (5%)	2 (5%)	0.99 *
Cancer	6 (6%)	4 (7%)	2 (7%)	0.99 *
Prolapse	4 (4%)	2 (3%)	2 (5%)	0.99 *
History of urinary stones	10 (9%)	10 (16%)	-	<0.01
At least one UTI in the last year	26 (29%)	15 (28%)	11 (31%)	0.78
No. of UTIs in the last year (if UTI)	1 (1–2)	1 (1–2)	1 (1–4)	0.21
Current UTI				
Number of days with symptomatic UTI before ED visit, median (IQR, 25–75%)	2 (1–5)	2 (1–5)	2 (1–4)	0.62
Symptoms, *n* (%)				
Algiuria	34 (34%)	17 (29%)	17 (42%)	0.21
Dysuria	50 (50%)	28 (48%)	22 (54%)	0.60
Pollakiuria	44 (44%)	17 (29%)	27 (66%)	<0.01
New urinary incontinence	7 (7%)	5 (9%)	2 (5%)	0.69 *
Macroscopic haematuria	11 (11%)	9 (16%)	2 (5%)	0.12 *
Flank pain	66 (67%)	41 (71%)	25 (61%)	0.31
Fever (≥38 °C)	74 (75%)	45 (78%)	29 (71%)	0.44
Vomiting	37 (37%)	24 (41%)	13 (32%)	0.33
Chills	18 (18%)	11 (19%)	7 (17%)	0.99 *
Clinical, biological evaluation				
Haemodynamic instability, *n* (%)	16 (15%)	11 (18%)	5 (11%)	0.42 *
Low systolic blood pressure (<100 mmHg)	21 (20%)	13 (21%)	8 (18%)	0.68 *
Low systolic blood pressure (<90 mmHg)	9 (8%)	8 (13%)	1 (2%)	0.08 *
Tachycardia (>100 bpm)	35 (33%)	19 (31%)	16 (36%)	0.59
Skin mottling	2 (2%)	2 (4%)	0 (0%)	0.51 *
eGFR (MDRD) ≤ 40 mL/min/1.73 m2	10 (10%)	9 (15%)	1 (2%)	0.04 *
urinary pH ≥ 7.0	11 (10%)	9 (15%)	2 (4%)	0.11 *
Gram-negative bacteria, *n* (%)	82 (80%)	45 (76%)	37 (84%)	0.46 *
*E. coli*	75 (73%)	42 (71%)	33 (75%)	0.67
*Morganella* spp.	1 (1%)	1 (2%)	-	0.99 *
*Klebsiella* spp.	6 (6%)	2 (3%)	4 (9%)	0.39 *
*Citrobacter* spp.	1 (1%)	1 (2%)	-	0.99 *
*Pseudomonas* spp.	-	-	-	-
*Gram-positive bacteria*, *n* (%)	7 (7%)	5 (8%)	2 (5%)	0.99 *
*Enterococcus* spp.	4 (4%)	4 (7%)	-	0.13 *
*Streptococcus* spp.	1 (1%)	-	1 (2%)	0.43 *
*Staphylococcus* spp.	1 (1%)	1 (2%)	-	0.99 *
*Aerococcus* spp.	2 (2%)	-	2 (5%)	0.18 *
In- and outpatient management				
Orientation after ED visit, *n* (%) Ambulatory Short stay unit (<24 h) Hospital	46 (46%) 5 (5%) 50 (49%)	22 (38%) 3 (5%) 33 (57%)	24 (56%) 2 (5%) 17 (40%)	0.21 *
Type of antibiotics treatment, *n* (%) Ceftriaxone Other	95 (89%) 12 (11%)	57 (92%) 5 (8%)	42 (84%) 7 (16%)	0.35 *
Persistent fever after 72 h, *n* (%)	9 (8%)	7 (11%)	2 (4%)	0.29 *
Need to change antibiotics, *n* (%)	14 (14%)	9 (15%)	5 (11%)	0.77 *
Length of hospital stay (if admitted), days, median (IQR, 25–75%)	5 (3–7)	5 (3–7)	5 (3–7)	0.67
Stay in intensive care unit (ICU), *n* (%)	2 (2%)	1 (2%)	1 (2%)	0.99 *
Intra-hospital death, *n* (%)	1 (1%)	1 (2%)	-	0.99 *

* Fisher’s exact test. IQR = interquartile range; UTI = urinary tract infection; ED = emergency department.

**Table 2:** Compliance with two different clinical rules indicating upper urinary tract imaging. Values are numbers (per cent; 95% confidence interval).

**Initial Imaging (≤24 h) and Recommendation Based on Van Nieuwkoop’s Study Criteria**			
	**Imaging Recommended** **(*n* = 28)**	**Imaging Not Recommended ** **(*n* = 72)**	***p*** -Value *
Imaging performed (*n* = 49)	23 (82%; 95%CI: 63–93%)	26 (36%; 95%CI: 26–48%)	<0.01
Imaging not performed (*n* = 51)	5 (18%; 95%CI: 7–37%)	46 (64%; 95%CI: 52–74%)	
**Early (≤24 h) and Delayed Imaging (>24 h) and European Association of Urology Recommendation**			
	**Imaging Recommended ** **(*n* = 44)**	**Imaging Not Recommended ** **(*n* = 56)**	***p*** **-Value**
Imaging performed (*n* = 60)	34 (77%; 95%CI: 62–87%)	26 (46%; 95%CI: 34–60%)	<0.01
Imaging not performed (*n* = 40)	10 (23%; 95%CI: 12–38%)	30 (54%; 95%CI: 40–66%)	

* Fisher’s exact test.

**Table 3:** Factors associated with clinically relevant abnormal findings on imaging among patients undergoing upper urinary tract imaging or an uneventful clinical course and no imaging (*n* = 99).

	OR (95% CI)	Se	Sp	PPV	NPV	AUC
Men	2.0 (0.7–6.1)	38 (15–65)	77 (67–86)	24 (9–45)	87 (77–93)	0.57 (0.44–0.71)
Older than median (>50 years)	1.5 (0.5–4.3)	63 (35–85)	48 (36–58)	19 (9–31)	87 (73–95)	0.55 (0.42–0.68)
Diabetes	0.1 (0.1–6.4)	0 (0–21)	96 (89–99)	0 (0–71)	82 (73–90)	0.48 (0.46–0.51)
Under immunosuppressive treatment	1.1 (0.1–7.5)	6 (2–30)	94 (87–98)	17 (4–64)	84 (75–91)	0.51 (0.43–0.57)
UTI in the last year (binary)	0.9 (0.3–2.9)	27 (8–55)	70 (58–81)	16 (4–36)	82 (70–91)	0.49 (0.36–0.62)
Structural urinary tract anomalies *	5.7 (1.8–18.2)	44 (20–70)	88 (79–94)	41 (18–67)	89 (80–95)	0.66 (0.53–0.79)
History of urinary stones	11.8 (3.0–46.5)	38 (15–65)	95 (88–99)	60 (26–88)	89 (80–95)	0.66 (0.54–0.79)
Gross haematuria	5.9 (1.6–22.1)	33 (12–62)	92 (84–97)	46 (17–77)	88 (79–94)	0.63 (0.51–0.76)
Flank pain	1.2 (0.3–4.2)	73 (45–92)	30 (17–46)	27 (14–43)	77 (50–93)	0.52 (0.38–0.65)
Nausea/vomiting	2.0 (0.7–5.9)	53 (27–79)	64 (52–74)	22 (10–39)	88 (76–95)	0.59 (0.44–0.73)
Haemodynamic instability **	0.8 (0.1–3.8)	13 (2–38)	86 (76–92)	14 (2–43)	84 (74–91)	0.49 (0.40–0.58)
Urinary pH ≥ 7.0	1.2 (0.1–5.3)	13 (2–38)	89 (80–95)	18 (2–52)	84 (74–91)	0.51 (0.42–0.60)
Nitrite (spot)	0.7 (0.2–1.9)	38 (15–65)	52 (40–64)	14 (5–28)	80 (66–90)	0.44 (0.31–0.58)
Haematuria (>15 red cells)	1.1 (0.4–3.2)	47 (21–73)	54 (42–67)	18 (8–34)	82 (68–92)	0.51 (0.36–0.65)
GFR < 40 mL/min/1.73 m^2^	2.3 (0.6–9.6)	19 (4–46)	91 (82–96)	30 (7–65)	85 (75–92)	0.55 (0.45–0.65)
Persistent fever > 72 h	0.1 (0.1–2.8)	0 (0–21)	92 (84–97)	0 (0–41)	83 (73–90)	0.46 (0.43–0.49)
Prediction rules						
Van Nieuwkoop’s criteria	5.4 (1.8–16.3)	63% (35–85%)	76% (65–85%)	36% (19–56%)	91% (81–97%)	0.70 (0.56–0.83)
EAU guidelines criteria	2.6 (0.9–7.5)	63% (35–85%)	61% (49–72%)	25% (13–41%)	89% (77–96%)	0.62 (0.48–0.75)

* Structural urogenital anomalies were considered when any congenital urological malformation, neurogenic bladder, benign prostatic hyperplasia, urinary cancer or urogenital prolapse was present, ** shock index < 1, blood pressure < 90 mmHg systolic or <65 mean blood pressure, or skin mottling. AUC = area under the receiver operating curve, EAU = European Association of Urology, GFR = glomerular filtration rate, Se = sensibility, Sp = specificity, PPV = positive predictive value, NPV = negative predictive value, OR = odds ratio, UTI = urinary tract infection.

## Data Availability

Data are available on request (Gregor.john@rhne.ch).

## Supplementary Materials

The following supporting information can be downloaded at: https://www.mdpi.com/article/10.3390/idr16020015/s1, Figure S1: Receiver Operating Characteristic (ROC) curves of individual factors associated with clinically relevant abnormal findings; Table S1: Radiological findings for the total study population that underwent upper urinary tract imaging, Table S2: Factors associated with clinically relevant abnormal findings (sensitivity analysis).
