# Supplementary material for: Radiological Explorations of Patients with Upper or Febrile Urinary Tract Infection
Source: Infect Dis Rep. 2024 Feb 23;16(2):189–99. doi: 10.3390/idr16020015 (PMC10961809; doi:10.3390/idr16020015)
Supplement: Supplementary file 1 [file idr-16-00015-s001.zip › idr-2857094-supplementary.pdf]

Radiological explorations of patients with upper or febrile urinary tract infection

Katia Vanolli, Mike Libasse Jost, Olivier Clerc, Daniel Genné, Gregor John

APPENDIX

Table S1: Radiological findings for the total study population that underwent upper urinary tract imaging p 2-3

Table S2: Factors associated with clinically relevant abnormal findings (sensitivity analysis) p 4

Figure S1: Receiver Operating Characteristic (ROC) curves of individual factors associated with clinically relevant abnormal findings p 5

**Table S1.** Radiological findings for the total study population that underwent upper urinary tract imaging.							
	**Total** N = 62	**Van Nieuwkoop's criteria**			**EAU criteria**		
		positive n = 25	negative n = 35	*p*-value	positive n = 34	negative n = 26	*p*-value
**Clinically relevant findings***	**15**	**9 (60%)**	**6 (40%)**	**0.13†**	**9 (60%)**	**6 (40%)**	**0.99†**
**Urgent urological disorder**	**8**	**4 (50%)**	**4 (50%)**	**0.71†**	**4 (50%)**	**4 (50%)**	**0.72†**
- Pyonephrosis	5	-	-		-	-	
- Other causes of obstructive pyelonephritis (e.g. tumour, urinary retention of any cause)	4	-	-		-	-	
- Obstructive lithiasis	4	-	-		-	-	
- Renal abscess	1	-	-		-	-	
- Emphysematous pyelonephritis	-	-	-		-	-	
**Non-urgent urological disorder**	**9**	**5 (56%)**	**4 (44%)**	**0.47†**	**5 (56%)**	**4 (44%)**	**0.99†**
- Non-obstructive lithiasis	7	-	-		-	-	
- Non-obstructive ureteropyelonephritis	2	-	-		-	-	
- Entero- or colovesical fistula	1	-	-		-	-	
- Urologic malignancy	-	-	-		-	-	
- Ureteropelvic junction stenosis	-	-	-		-	-	
**Obstructive and non-obstructive stones**	**9**	**5 (71%)**	**2 (29%)**	**0.12†**	**5 (71%)**	**2 (29%)**	**0.69†**
**Clinically irrelevant findings***	**47**	**16 (36%)**	**29 (64%)**	**0.13**	**25 (56%)**	**20 (44%)**	**0.99†**
**Normal imaging**	**14**	**7 (33%)**	**14 (67%)**	**0.42†**	**11 (52%)**	**10 (48%)**	**0.62**
**Minimal urological findings**	**28**	**11 (39%)**	**17 (61%)**	**0.73**	**15 (54%)**	**13 (46%)**	**0.65**
- Signs of acute pyelonephritis	13	-	-		-	-	
- Renal cyst	7	-	-		-	-	
- Benign prostate hyperplasia (without urinary retention)	5	-	-		-	-	
- Kidney atrophy	5	-	-		-	-	
- Vesical prolapse	1	-	-		-	-	
- Bladder diverticula	1	-	-		-	-	
- Bifid ureter	1	-	-		-	-	
**Incidental non-urological findings**	**11**	**3 (27%)**	**8 (73%)**	**0.33†**	**4 (37%)**	**7 (64%)**	**0.18†**
- Diverticulosis	4	-	-		-	-	

- Cholelithiasis	2	-	-		-	-	
- Ovarian cyst	1	-	-		-	-	
- Inguinal hernia	1	-	-		-	-	
- Dysmorphic liver	1	-	-		-	-	
- Bone metastasis	1	-	-		-	-	
- Significant coprostasis	1	-	-		-	-	
- Ascites	1	-	-		-	-	
* Since patients can have several abnormal findings, anomaly totals are not cumulative; † Fischer’s exact test							

Table S2: Factors associated with clinically relevant abnormal findings (sensitivity analysis). The analyses were restricted to patients who underwent upper urinary tract imaging (N = 62).

	**OR (95% CI)**	**Se**	**Sp**	**PPV**	**NPV**	**AUC**
**Men**	1.5 (0.4–5.0)	33 (12–62)	75 (60–86)	29 (10–56)	78 (63–89)	0.54 (0.40–0.68)
**Older than median (> 50 years)**	1.3 (0.4–4.1)	60 (32–84)	47 (32–62)	27 (13–44)	79 (59–92)	0.53 (0.39–0.68)
**Diabetes**	0.1 (0.1–5.7)	0 (0–22)	95 (84–99)	0 (0–84)	73 (60–84)	0.48 (0.44–0.51)
**Under immunosuppressive treatment**	1.1 (0.1–8.2)	7 (2–32)	94 (83–99)	25 (1–91)	76 (63–86)	0.50 (0.43–0.58)
**UTI in the last year (bin)**	1.1 (0.3–3.9)	29 (8–58)	73 (56–85)	27 (8–55)	74 (58–87)	0.51 (0.36–0.65)
**Structural urinary tract anomalies***	4.6 (1.2–16.8)	40 (16–68)	87 (74–95)	50 (21–79)	82 (69–91)	0.64 (0.50–0.77)
**History of urinary stones**	7.2 (1.8–29.0)	40 (16–68)	92 (80–98)	60 (26–88)	83 (70–92)	0.66 (0.52–0.79)
**Gross haematuria**	4.9 (1.2–20.2)	33 (12–62)	91 (78–97)	56 (21–86)	80 (66–90)	0.62 (0.49–0.75)
**Flank pain**	1.2 (0.3–4.2)	73 (45–92)	30 (17–46)	27 (14–43)	77 (50–93)	0.52 (0.38–0.65)
**Nausea/vomiting**	1.9 (0.6–6.2)	53 (27–78)	63 (47–77)	33 (16–55)	79 (62–91)	0.58 (0.43–0.73)
**Haemodynamic instability****	0.6 (0.1–3.1)	13 (2–41)	81 (67–91)	18 (2–52)	75 (60–86)	0.47 (0.37–0.58)
**Urinary PH ≥ 7.0**	0.3 (0.1–2.3)	7 (1–32)	83 (69–92)	11 (1–48)	73 (59–84)	0.45 (0.36–0.53)
**Nitrite (spot)**	0.8 (0.2–2.6)	40 (16–68)	55 (39–70)	24 (9–45)	72 (53–86)	0.47 (0.32–0.62)
**Haematuria (> 15 red cells)**	0.6 (0.2–2.1)	43 (18–71)	46 (31–63)	21 (8–41)	70 (50–86)	0.45 (0.30–0.60)
**GFR < 40** ml/min/1.73 m^2	1.7 (0.4–7.2)	20 (4–48)	87 (74–95)	33 (7–70)	77 (63–88)	0.54 (0.42–0.65)
**Persistent fever > 72 h**	0.1 (0.1–1.6)	0 (0–22)	85 (72–94)	0 (0–41)	73 (59–84)	0.43 (0.37–0.48)
**Prediction rules**						
**Van Nieuwkoop's study criteria**	2.7 (0.8–8.7)	60% (32–84%)	64% (49–78%)	36% (18–58%)	83% (66–93%)	0.62 (0.48–0.77)
**EAU guidelines criteria**	1.2 (0.4–3.8)	60% (32–84%)	44% (30–60%)	27% (13–44%)	77% (56–91%)	0.52 (0.37-0.67)

* Structural urogenital anomalies were considered when any congenital urological malformation, neurogenic bladder, benign prostatic hyperplasia, urinary cancer or urogenital prolapse was present, ** Shock index < 1, blood pressure < 90 mmHg systolic or < 65 mean blood pressure, or skin mottling.

AUC = area under the receiver operating curve, EAU = European Association of Urology, GFR = glomerular filtration rate, Se = sensibility, Sp = specificity, PPV = positive predictive value, NPV = negative predictive value, OR = odds ratio, UTI = urinary tract infection

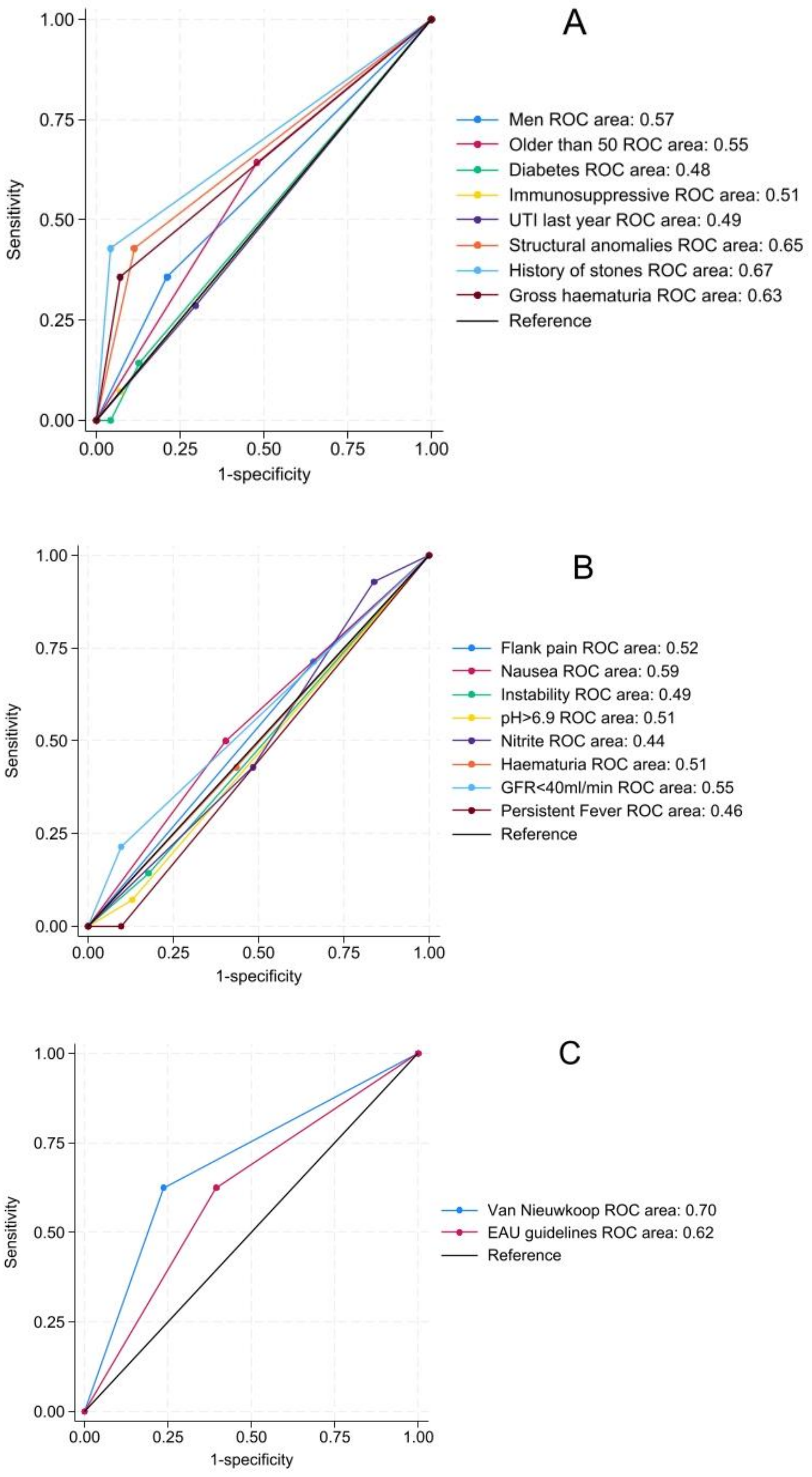

Figure S1: Receiver Operating Characteristic (ROC) curves of individual factors associated with clinically relevant abnormal findings (A, B), and of the Van Nieuwkoop and European Association of Urology (EAU) guidelines. Other information can be found in the main manuscript (Table3).
